# Identification of a Novel p.Q1772X *ANK1* Mutation in a Korean Family with Hereditary Spherocytosis

**DOI:** 10.1371/journal.pone.0131251

**Published:** 2015-06-24

**Authors:** Joo Hyung Han, Seung Kim, Hoon Jang, So Won Kim, Min Goo Lee, Hong Koh, Ji Hyun Lee

**Affiliations:** 1 Yonsei University College of Medicine, Seoul, Korea; 2 Department of Pediatrics, Severance Children’s Hospital, Yonsei University College of Medicine, Seoul, Korea; 3 Department of Chemistry, Yonsei University, Seoul, Korea; 4 Department of Pharmacology and PharmacoGenomics Research Center, Inje University College of Medicine, Busan, Korea; 5 Department of Clinical Pharmacology, Inje University, Busan Paik Hospital, Busan, Korea; 6 Department of Pharmacology, Pharmacogenomic Research Center for Membrane Transporters, Brain Korea 21 PLUS Project for Medical Sciences, Severance Biomedical Science Institute, Yonsei University College of Medicine, Seoul, Korea; 7 Department of Oral Biology, Yonsei University College of Dentistry, Seoul, Korea; University of Heidelberg, GERMANY

## Abstract

Hereditary spherocytosis (HS), a common form of inherited hemolytic anemia, is a heterogeneous group of disorders with regard to clinical severity, protein defects, and mode of inheritance. Causal mutations in at least five genes have been reported so far. Because multiple genes have been associated with HS, clinical genetic testing that relies on direct sequencing will be a challenge. In this study, we used whole exome sequencing to identify a novel nonsense mutation in *ANK1* (p.Q1772X, NM_020476) that resulted in a truncated protein in a Korean patient with HS. Sanger sequencing confirmed the two affected individuals in the patient’s family were heterozygous for the mutation. This is the first report of a Korean family that carries an *ANK1* mutation responsible for HS. Our results demonstrate that next generation sequencing is a powerful approach for rapidly determining the genetic etiology of HS.

## Introduction

Hereditary spherocytosis (HS) is a common form of inherited hemolytic anemia characterized by hemolysis, jaundice, splenomegaly, and gallstones [1]. HS has a wide spectrum of disease severity, and its prevalence is 1 in 2,000 in people of Northern European descent [2, 3]. The prevalence of HS in people of other ethnic backgrounds is much lower. To date, it has been shown that mutations in several genes, including those that encode spectrin, ankyrin, band 3 protein, protein 4.2, and other erythrocyte membrane proteins, are associated with this condition [4].

Erythroid ankyrin (ANK1) is a major protein in erythrocytes; it is involved in the linkage of transmembrane proteins and the cell membrane skeleton via spectrin, band 3, and band 4.2 proteins [5]. Multiple isoforms of ankyrin are expressed in a tissue-specific, developmentally-regulated manner [6]. Ankyrins are typically composed of three structural domains: an N-terminal domain containing multiple ankyrin repeats; a center region containing a spectrin-binding domain; and a C-terminal regulatory domain, which is the least conserved [7, 8]. *ANK1* mutations have been identified in approximately half of all patients with HS. They most commonly exhibit an autosomal dominant pattern of inheritance, but they may also display an autosomal recessive pattern. Disease severity ranges from mild to severe depending on the extent of the membrane defect [9, 10].

Gilbert’s syndrome (GS) is a very common cause of unconjugated hyperbilirubinemia associated with *UGT1A1* mutations [11]. GS does not usually require treatment, and its clinical significance is low, but this condition can be comorbid with other causes of hyperbilirubinemia, giving rise to diagnostic confusion. Although HS is a well-established disorder, some cases are comorbid with GS [12–14].

Recent advances in next generation sequencing (NGS) technology have led to a paradigm shift, leading the field of genetic testing away from Sanger sequencing [15]. Cost-effective, high-throughput NGS has led to the clinical implementation of whole exome sequencing (WES). WES has contributed greatly to the discovery of novel mutations responsible for Mendelian diseases [16]. It is widely employed as a diagnostic method, as it allows researchers to screen the entire coding regions of genes [17, 18]. In this study, we identified a novel *ANK1* mutation responsible for HS in a Korean family via WES. This demonstrates that WES is an effective method both for identifying novel causal mutations and for diagnosing additional disease cases.

## Materials and Methods

### Subjects

The Department of Hematology at Severance Hospital referred a 19-year-old male patient to the Gastroenterology Department due to hyperbilirubinemia. The patient had neonatal hyperbilirubinemia and was diagnosed with HS at 3 months of age. The diagnosis was made based on spherocytosis seen in a peripheral blood smear. His mother had received a cholecystectomy and splenectomy roughly 30 years before without knowing the reason for the operations. At 6 years old, the patient underwent a splenectomy due to anemia and splenomegaly. After the splenectomy, his hemoglobin levels improved to normal range and his bilirubin levels decreased dramatically. Mild hyperbilirubinemia persisted for several years, and thus we performed genetic analyses and serologic tests upon the patient and his immediate family members to confirm the HS diagnosis and determine the cause of his persistent hyperbilirubinemia. Written informed consent was obtained from all participants. All clinical investigation has been conducted according to the principles expressed in the Declaration of Helsinki. The parents of the individual whose information appears in this manuscript have given written informed consent (as outlined in the PLOS consent form) to publish these case details. This study was approved by the Institutional Review Board of Severance Hospital, Yonsei University Health System (4-2010-0032).

### Genetic Testing

WES was conducted using the Agilent SureSelect Enrichment System, according to the manufacturer’s instructions. The patient’s exome DNA was captured using the SureSelect All Exon V4+UTRs kit (Agilent Technologies) and sequenced as paired-end 150 bp reads on the Illumina HiSeq 2500 platform (Illumina Inc.). Sequencing data are accessible at Sequence Read Archive (http://trace.ncbi.nlm.nih.gov/Traces/sra/, accession number SRA: SRR1713040). Sequence reads were aligned to human reference genome 19 (hg19) using Novoalign (v3.01.01) and re-aligned with the Genome Analysis Toolkit (v2.3.6). Duplicates were removed using Picard (v1.67). Variant calling was performed using the GATK Unified Genotyper (v2.3.6). Single nucleotide variants (SNVs) and small indels were annotated using ANNOVAR [19]. In addition, we rescreened all variants for splice sites, which are likely to be missed by conventional annotation programs. We estimated exons within ±2 bp from transcript isoforms downloaded from the UCSC Table Browser. A total of 10,944 variants were identified (10,246 SNVs and 698 indels). Based on the patient’s clinical history, variants in candidate genes linked to hyperbilirubinemia and hereditary spherocytosis were prioritized. The sequencing coverage and variants detected in these candidate genes are shown in S1 and S2 Tables, respectively. The average coverage for each exon in the spherocytosis genes that analyzed in this study are shown in S1 Fig. To remove common variants, those with a minor allele frequency (MAF) >5% were filtered out; MAF was determined using an in-house Korean control WES dataset of 276 samples and the 1000 Genomes Project dataset. For the excluded mutations with a MAF >5%, additional screening was performed to identify those related to this relatively common disease. Sanger sequencing was performed on DNA samples obtained from immediate family members. Genomic DNA was extracted from the blood samples of the patient’s family members using a QIAamp DNA Blood Mini Kit (Qiagen, Valencia, CA, USA), and we designed Sanger validation primers using Primer3 (v4.0.0) software (http://bioinfo.ut.ee/primer3/). Primers were designed for the *ANK1* (forward, ANK1-F: 5’-TAAAGGAAGGGGATATGCTTAGCTCAGGG-3’, reverse, ANK1-R: 5’-TGTCTAAAAGGAATGAGACTGATGGGTGAC-3’) and *UGT1A1* (forward, UGT1A1-F: 5’-ATATATATATATAAGTAGGAGAGGGCGAAC-3’, reverse, UGT1A1-R: 5’-TATTTTCTTGTATGTTTTGATCACACGC-3’) genes. Sequencing was performed using an ABI 3730xl DNA Analyzer (Applied Biosystems).

## Results

### Clinical Features of a Korean Family with HS

The patient after splenectomy had no clinical symptoms or signs of HS. However mild indirect hyperbilirubinemia has persisted for more than 10 years, showing intermittent fluctuations (total bilirubin (TB) / direct bilirubin (DB), 1.3/0.6 to 7.5/0.8 mg/dL). In addition, mild reticulocytosis (reticulocyte index, 2.8%; absolute reticulocyte count, 133.7x103/μL) along with the blood smear are signs of HS. His mother also exhibited slightly elevated bilirubin levels (TB/DB, 1.3/0.4 mg/dL) without anemia. She only has limited clinical information, such as records of cholecystectomy and splenectomy. Neither his father nor elder brother had hyperbilirubinemia or anemia.

### Characterization of *ANK1* mutation

**Fig 1:**
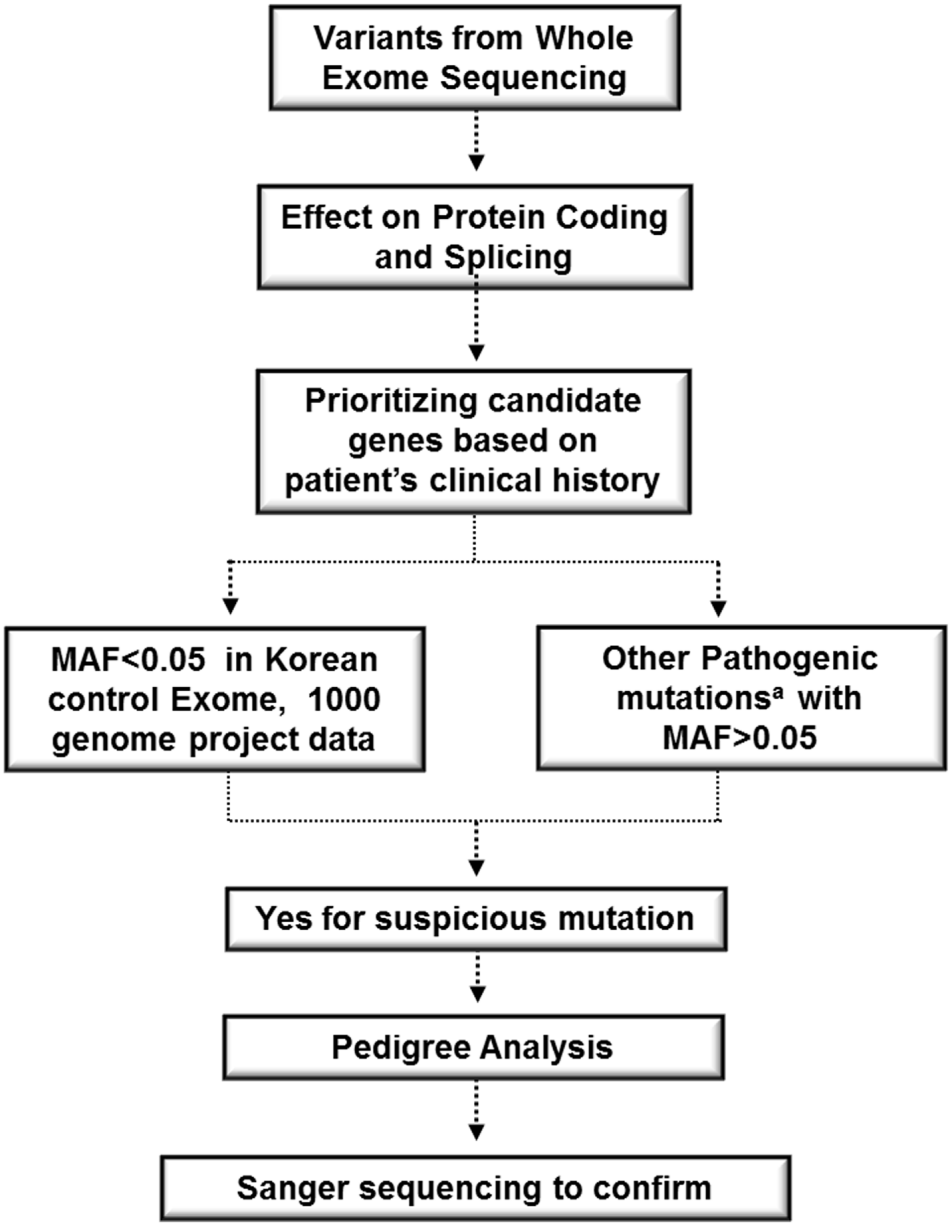
Workflow of whole exome sequencing bioinformatics analysis pipeline. ^a^The pathogenicity was evaluated based on current knowledge.

WES achieved 75.29X average depth-of-coverage; 99.5% and 96.6% of the exome sequence was covered at 1X and 10X, respectively. A total of 10,944 variants identified in the index patient were subjected to a process designed to discover pathogenic mutations (Fig 1). After applying filtration process from WES result, we identified two mutations that could be implicated in the patient’s phenotype. One was a nonsense variant in *ANK1* (c.C5314T, p.Q1772X, exon 39, NM_020476), and the other was a missense variant in *UGT1A1* (c.G211A, p.G71R, exon 1, NM_000463).

**Fig 2:**
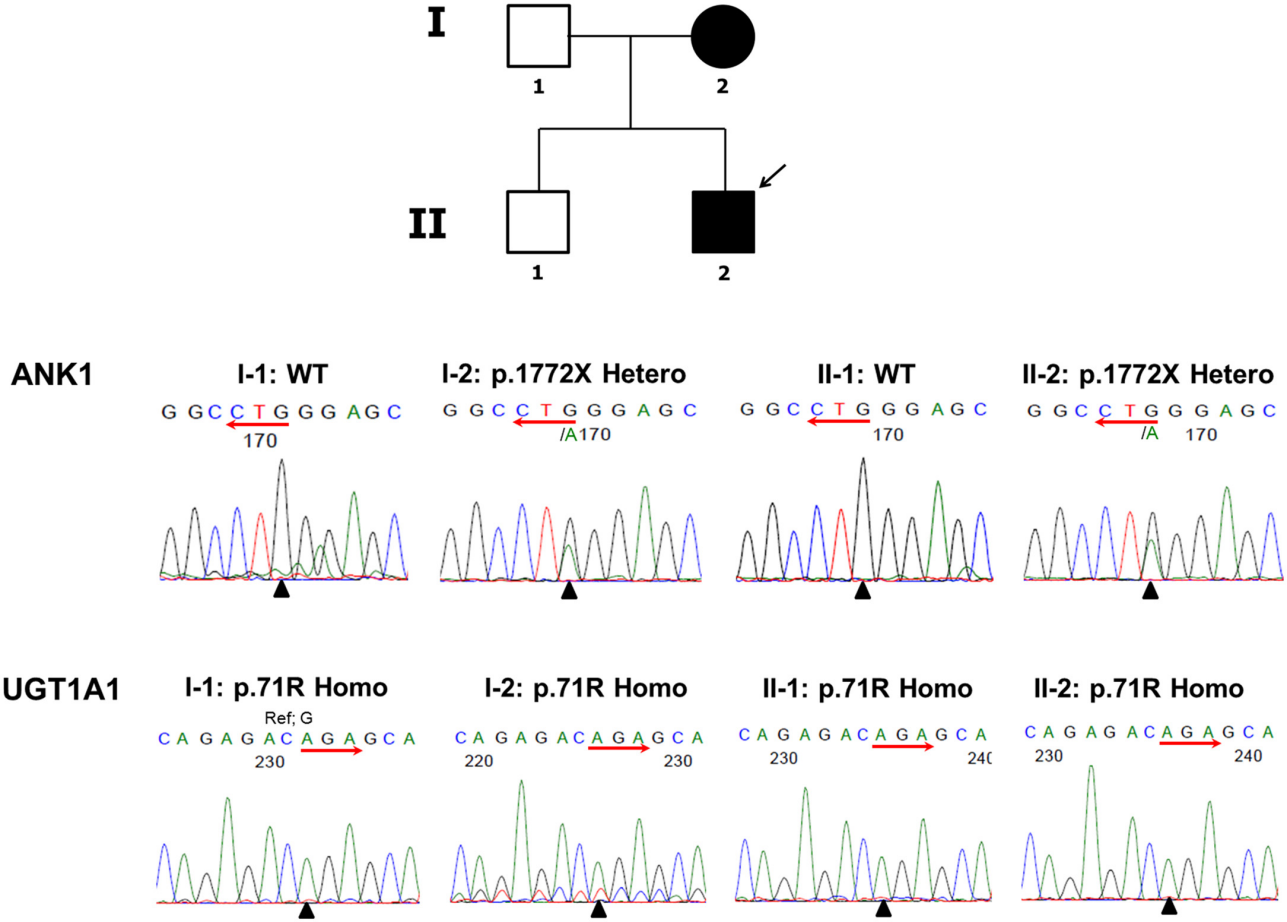
Pedigree of the family affected by hereditary spherocytosis (HS) and the mutations identified. A. Individuals affected with HS are indicated by a black filled circle (females) or square (males). The proband (II-2) is indicated by an arrow. B. Electropherograms indicate *ANK1* and *UGT1A1* mutations identified in the family.

Sanger sequencing of these loci was performed on all immediate family members. These results showed that the patient (II.2) and his mother (I.2) were heterozygous for the *ANK1* variant, while the patient’s father (I.1) and brother (II.1) had wild-type *ANK1* alleles (Fig 2A). The p.Q1772X variant is located in the C-terminal regulatory region of *ANK1* and results in premature termination of the Ankyrin 1 protein. This variant was not found in our in-house Korean control database, demonstrating its rarity. Additionally, a search of the Human Gene Mutation Database (http://www.hgmd.cf.ac.uk/) confirmed p.Q1772X as a novel mutation. By contrast, Sanger sequencing demonstrated all family members were homozygous for the other variant, p.G71R in *UGT1A1* (Fig 2B). This variant is known to be associated with GS and is especially prevalent among Korean and Japanese GS patients [20]. The allele frequency of this mutation was 16.4%, based on our in-house Korean control database.

## Discussion

Here, we describe a Korean family with two members affected by HS. WES of the index patient revealed a p.Q1772X *ANK1* mutation and a p.G71R *UGT1A1* variant. Sanger sequencing of these loci in the patient’s immediate family clarified the relationship between these variants and the Mendelian disease HS.

To date, 23 point mutations in *ANK1* have been identified among patients with HS [21]. Like the novel p.Q1772X mutation reported in this study, five have been nonsense mutations located in the protein’s regulatory domain. Deficient ankyrin protein levels due to nonsense mutations located in the regulatory region have previously been proposed as a cause of HS [22]. An *in vivo* functional study has also confirmed that a nonsense mutation in *ANK1* leads to the development of spherocytosis [23]. Our patient shows heightened sensitivity in an osmotic fragility test, which is a known pathological feature of HS caused by an *ANK1* mutation.

Genetic testing of all the index patient’s immediate family members revealed that both the patient and his mother were heterozygous for the p.Q1772X *ANK1* mutation. The patient’s mother reported having received a cholecystectomy and splenectomy, indicating a high possibility of spherocytosis despite her incomplete past medical history. Therefore, we were able to find a disease causing genetic variant of HS. This represents the first report correlating the p.Q1772X mutation in *ANK1* to HS in a Korean family.

All family members were homozygous for the p.G71R *UGT1A1* variant. The p.G71R variant, also known as UGT1A1*6, causes GS, along with UGT1A1*7, UGT1A1*27, UGT1A1*28, and UGT1A1*62 [24, 25]. However, only the index patient (II.2) and his mother (I.2) displayed mild hyperbilirubinemia, while the patient’s father (I.1) and brother (II.1) were normal. This incomplete penetrance of GS may result from a mild decrease in function attributable to UGT1A1*6. Relative to other GS causal variants, the effect of UGT1A1*6 on UGT1A1 enzyme activity is not very severe [26]. Therefore we conclude that the UGT1A1*6 variant is only a risk factor for hyperbilirubinemia, with heterogeneity in phenotypes observed among individuals.

Our study differs from other reports with respect to the way that pedigree analysis was conducted on coexisting GS and HS. Here, we were able to verify that the phenotypic variance resulted from the p.Q1772X mutation by comparing clinical characteristics of family members with and without the mutation. The two family members without the p.Q1772X *ANK1* mutation functioned as negative controls; this was possible because all family members shared the same *UGT1A1* genetic background, being homozygous for the p.G71R variant. Mild hyperbilirubinemia were observed in the proband (II.2) and his mother (I.2), related to the presence of the p.Q1772X *ANK1* mutation. Thus, layered on a homozygous p.G71R *UGT1A1* background, the additional *ANK1* mutation appeared to cause hyperbilirubinemia. In the future, further functional studies may verify the relative contribution and quantitative risk for hyperbilirubinemia associated with each variant.

In the genetic diagnosis of HS, Sanger sequencing or targeted capture sequencing would probably be sufficient, with no need for whole exome sequencing (WES). However, disorders with phenotypic or genetic heterogeneity and patients with overlapping symptoms are difficult to diagnose. The available clinical differential diagnostic tests may be time-consuming and costly. Moreover, it is difficult to select the target genes in these cases. WES provides insights into genetic diagnosis of these types of challenging cases. The case-patient described in this study has limited clinical information except for the elevated bilirubin level. In this case, WES is the most useful tool, as it investigates all possible genetic causes.

In summary, a novel nonsense mutation in *ANK1* (p.Q1772X) was identified in a Korean family affected by HS. The proband had spherocytosis combined with unconjugated hyperbilirubinemia. Five known candidate genes are associated with HS, all involved in erythrocyte cytoskeleton formation. In addition, more than 20 genes are related to hyperbilirubinemia and bilirubin metabolism. Thus, to pinpoint the mutation causing the patient’s symptoms, we combined WES with pedigree analysis. This study shows that WES is a time and cost-effective method of identifying causal mutations relative to classical genetic tests, especially when symptoms of multiple conditions and a large number of candidate genes are involved.

## Supporting Information

S1 Fig The average coverage for each exon in the spherocytosis genes that were analyzed in this study. The blue and red bars on the graph indicate 1X and 10X coverage, respectively. The green triangles show the mean depth for each exon.
(DOCX)

S1 Table The sequencing coverage of the known candidate genes linked to Hereditary Spherocytosis and to hyperbilirubinemia. (DOCX)

S2 Table Variants detected in HS and hyperbilirubinemia candidate genes. (DOCX)
