# Supplementary material for: Identification of a Novel p.Q1772X ANK1 Mutation in a Korean Family with Hereditary Spherocytosis
Source: PLoS One. 2015 Jun 24;10(6):e0131251. doi: 10.1371/journal.pone.0131251 (PMC4480973; doi:10.1371/journal.pone.0131251)

**S1-Fig.** The average coverage for each exon in the spherocytosis genes that were analyzed in this study. The blue and red bars on the graph indicate 1X and 10X coverage, respectively. The green triangles show the mean depth for each exon.


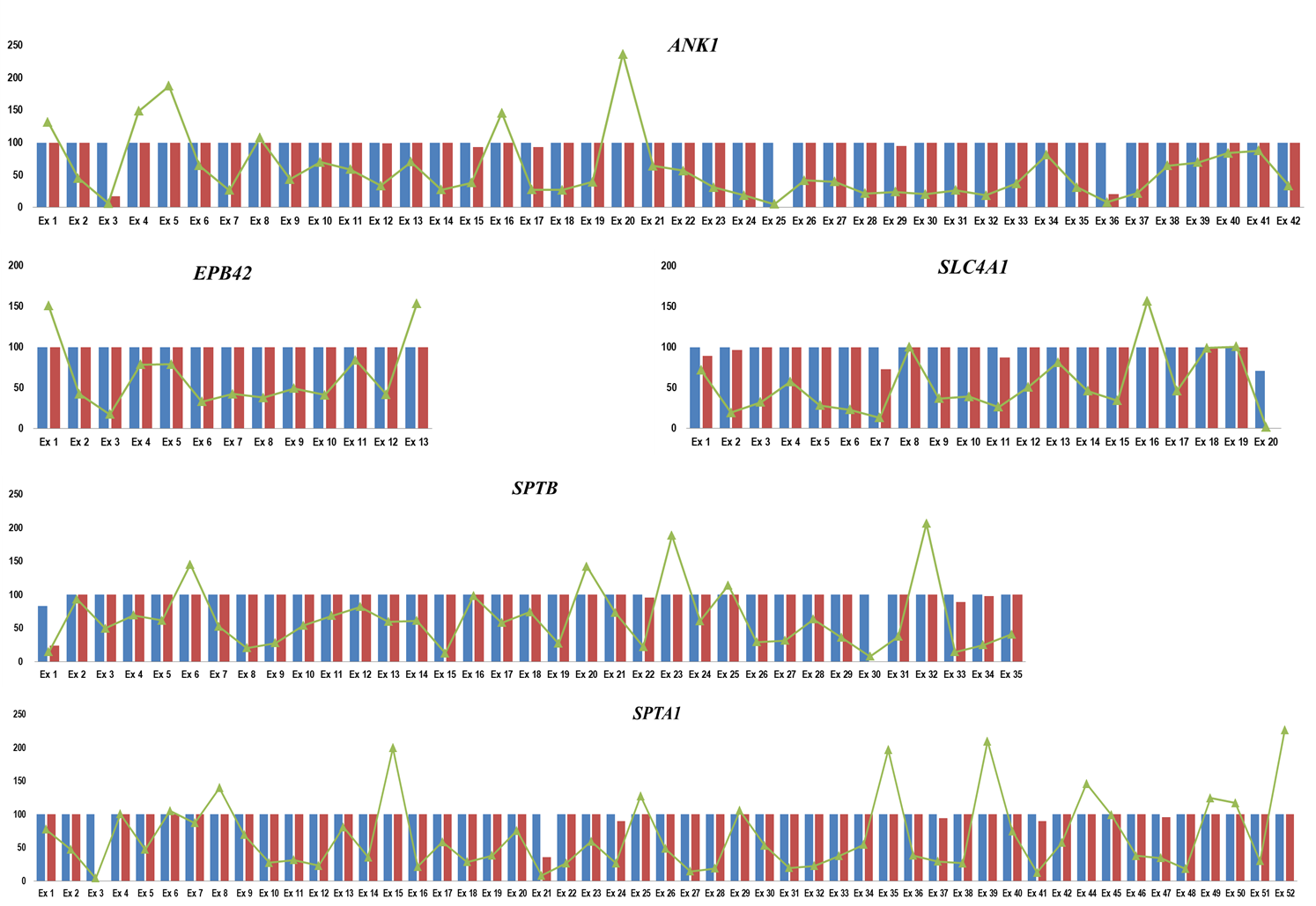

Supplement: S1 Fig — The blue and red bars on the graph indicate 1X and 10X coverage, respectively. The green triangles show the mean depth for each exon. (DOCX) [file pone.0131251.s001.docx]
