# Supplementary material for: Identification of a Novel p.Q1772X ANK1 Mutation in a Korean Family with Hereditary Spherocytosis
Source: PLoS One. 2015 Jun 24;10(6):e0131251. doi: 10.1371/journal.pone.0131251 (PMC4480973; doi:10.1371/journal.pone.0131251)
Supplement: S1 Table — (DOCX) [file pone.0131251.s002.docx]

**S1-Table.** The sequencing coverage of the known candidate genes linked to Hereditary Spherocytosis and to hyperbilirubinemia

| **Gene** | **Related diseases** | **Average depth-of-coverage** | **1X coverage** | **10X coverage** |
| --- | --- | --- | --- | --- |
| *ANK1* | Hereditary Spherocytosis | 57.7 | 100.0 | 93.3 |
| *EPB42* | Hereditary Spherocytosis | 65.5 | 100.0 | 100.0 |
| *SLC4A1* | Hereditary Spherocytosis | 53.2 | 98.5 | 92.3 |
| *SPTB* | Hereditary Spherocytosis | 63.5 | 99.5 | 94.5 |
| *SPTA1* | Hereditary Spherocytosis | 68.7 | 100.0 | 96.8 |
| *UGT1A1* | Hyperbilirubinemia | 99.0 | 100.0 | 100.0 |
| *ABCC2* | Hyperbilirubinemia | 59.1 | 100.0 | 98.0 |
| *GSTA1* | Hyperbilirubinemia | 18.8 | 85.7 | 73.1 |
| *SLCO1B1* | Hyperbilirubinemia | 67.9 | 90.6 | 89.5 |
| *SLCO1B3* | Hyperbilirubinemia | 44.8 | 85.0 | 69.5 |
| *ATP8B1* | Hyperbilirubinemia | 60.2 | 96.4 | 92.8 |
| *ABCB11* | Hyperbilirubinemia | 55.0 | 96.4 | 94.1 |
| *ABCB4* | Hyperbilirubinemia | 50.7 | 100.0 | 93.6 |
| *G6PD* | Hyperbilirubinemia | 28.1 | 100.0 | 82.0 |
