# Supplementary material for: Identification of a Novel p.Q1772X ANK1 Mutation in a Korean Family with Hereditary Spherocytosis
Source: PLoS One. 2015 Jun 24;10(6):e0131251. doi: 10.1371/journal.pone.0131251 (PMC4480973; doi:10.1371/journal.pone.0131251)
Supplement: S2 Table — (DOCX) [file pone.0131251.s003.docx]

**S2-Table.** Variants detected in HS and hyperbilirubinemia candidate genes

| **Coordinate** | **Gene** | **Ref/Var** | **AA_**  **change** | **II-2 genotype** | **dbSNP** | **SIFT** | **PolyPhen** | **1000g_Total VAF** | **1000g_ASN**  **VAF** | **Korean**  **VAF** |
| --- | --- | --- | --- | --- | --- | --- | --- | --- | --- | --- |
| chr1:158584091 | *SPTA1* | A/G | p.I2265T | het-var | rs952094 | tolerated | benign | A:0.4504 | G:0.60 | 0.65 |
| chr1:158607935 | *SPTA1* | T/G | p.K1693Q | het-var | rs857725 | tolerated | benign | G:0.2759 | G:0.40 | 0.42 |
| chr1:158612236 | *SPTA1* | A/G | p.C1568R | het-var | rs863931 | tolerated | benign | A:0.3760 | G:0.61 | 0.65 |
| chr1:158619728 | *SPTA1* | A/C | p.S1163A | hom-var | rs2482965 | tolerated | benign | A:0.0032 | - | 1.00 |
| chr2:234669144 | *UGT1A1* | G/A | p.G71R | hom-var | rs4148323 | Tolerated | possibly damaging | A:0.0523 | A:0.17 | 0.16 |
| chr8:41525865 | *ANK1* | G/A | p.Q1813X | het-var | - | - | - | - | - | 0.00 |
| chr10:101544447 | *ABCC2* | A/T | p.Y39F | hom-var | rs927344 | Tolerated | benign | A:0.0041 | - | 1.00 |
| chr12:21329738 | *SLCO1B1* | A/G | p.N130D | hom-var | rs2306283 | Tolerated | benign | A:0.4050 | G:0.75 | 0.70 |
| chr12:21331549 | *SLCO1B1* | T/C | p.V174A | het-var | rs4149056 | deleterious | Probably damaging | C:0.1230 | C:0.13 | 0.16 |
| chr17:42338998 | *SLC4A1* | T/G | p.D38A | het-var | rs5035 | tolerated | benign | G:0.0390 | G:0.08 | 0.07 |
| chr18:55317676 | *ATP8B1* | C/T | p.A1152T | hom-var | rs222581 | tolerated | benign | C:0.0005 | - | 1.00 |
| chr18:55336626 | *ATP8B1* | A/G | p.M674T | het-var | rs35470719 | tolerated | benign | G:0.0142 | G:0.05 | 0.06 |
| chr18:55342156 | *ATP8B1* | T/C | p.I577V | het-var | rs3745078 | tolerated | benign | C:0.0142 | C:0.05 | 0.06 |
| chr18:55373767 | *ATP8B1* | G/C | p.H78Q | het-var | rs3745079 | tolerated | benign | C:0.0147 | C:0.05 | 0.06 |

Abbreviation: AA, Amino Acid; het-var, hetero variant; hom-var, homo variant; ASN, Asian; VAF, variant allele frequency
